# (*Z*)-Methyl 3-(2,4-dichloro­phen­yl)-3-hy­droxy­acrylate

**DOI:** 10.1107/S1600536811051014

**Published:** 2011-12-03

**Authors:** Le-Xing Xu, Xiao-Guang Bai, Ju-Xian Wang, Yu-Cheng Wang

**Affiliations:** a Institute of Medicinal Biotechnology, Chinese Academy of Medical Sciences and Peking Union Medical College, Beijing 100050, People’s Republic of China

## Abstract

The mol­ecular structure of the title compound, C_10_H_8_Cl_2_O_3_, exists in a *cis*-enol form, which is stabilized by a strong intra­molecular O—H⋯O hydrogen bond. In the crystal, C—H⋯O inter­actions generate zigzag chains along the *c* axis which are, in turn, linked by further C—H⋯O inter­actions into sheets parallel to (100).

## Related literature

For the synthesis of the title compound, see: Wu *et al.* (1997 ▶). For related structures, see: Mei & Huang (2007 ▶); Zheng, Fan *et al.* (2007 ▶); Zheng, Zheng *et al.* (2007 ▶). For the coordination properties of similar compounds, see: Nakamoto *et al.* (1970 ▶); Ma *et al.* (1999 ▶); Yoshida *et al.*(2005 ▶).
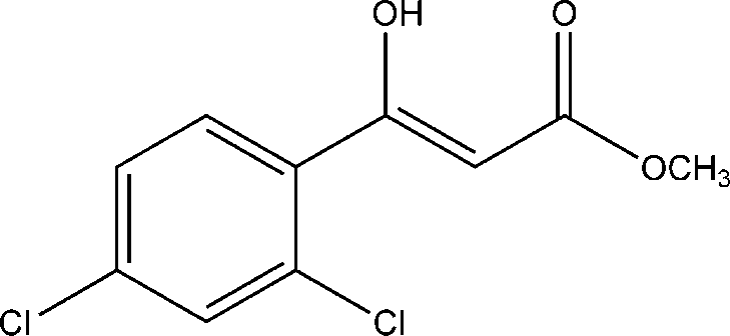


## Experimental

### 

#### Crystal data

C_10_H_8_Cl_2_O_3_
*M* *_r_* = 247.06
Monoclinic, 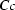

*a* = 15.889 (3) Å
*b* = 3.8242 (8) Å
*c* = 18.204 (4) Å
β = 108.18 (3)°
*V* = 1050.9 (4) Å^3^
*Z* = 4
Mo *K*α radiation
μ = 0.60 mm^−1^
*T* = 294 K
0.25 × 0.20 × 0.15 mm

#### Data collection

Rigaku SCXmini diffractometer
5011 measured reflections
2371 independent reflections
2170 reflections with *I* > 2σ(*I*)
*R*_int_ = 0.030

#### Refinement

*R*[*F* ^2^ > 2σ(*F* ^2^)] = 0.034
*wR*(*F* ^2^) = 0.086
*S* = 1.07
2371 reflections
139 parameters
2 restraints
H-atom parameters constrained
Δρ_max_ = 0.17 e Å^−3^
Δρ_min_ = −0.18 e Å^−3^
Absolute structure: Flack (1983 ▶), 1177 Friedel pairs
Flack parameter: 0.07 (6)

### 

Data collection: *CrystalClear* (Rigaku, 2005 ▶); cell refinement: *CrystalClear*; data reduction: *CrystalClear*; program(s) used to solve structure: *SHELXS97* (Sheldrick, 2008 ▶); program(s) used to refine structure: *SHELXL97* (Sheldrick, 2008 ▶); molecular graphics: *SHELXTL/PC* (Sheldrick, 2008 ▶); software used to prepare material for publication: *SHELXTL/PC*.

## Supplementary Material

Crystal structure: contains datablock(s) I, global. DOI: 10.1107/S1600536811051014/lr2037sup1.cif

Structure factors: contains datablock(s) I. DOI: 10.1107/S1600536811051014/lr2037Isup2.hkl

Supplementary material file. DOI: 10.1107/S1600536811051014/lr2037Isup3.cml

Additional supplementary materials:  crystallographic information; 3D view; checkCIF report

## Figures and Tables

**Table 1:** Hydrogen-bond geometry (Å, °)

*D*—H⋯*A*	*D*—H	H⋯*A*	*D*⋯*A*	*D*—H⋯*A*
O1—H1⋯O2	0.82	1.87	2.592 (3)	146
C3—H3⋯O2^i^	0.93	2.48	3.356 (3)	157
C10—H10*B*⋯O1^ii^	0.96	2.57	3.492 (3)	162

Symmetry codes: (i) 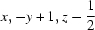; (ii) 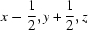.

**Fig. 1.**
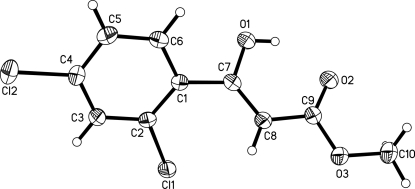
The molecular structure of the title compound. Displacement ellipsoids are drawn at the 30% probability level.

**Fig. 2.**
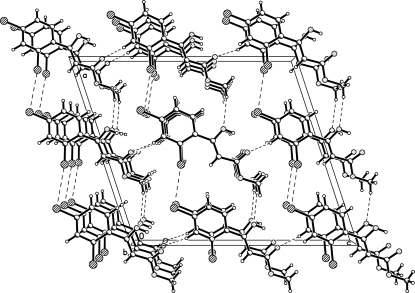
Packing diagram of the title compound viewed along the b axis. Hydrogen bonds are shown as dashed lines.

## supplementary crystallographic information

### Comment

1,3-Diketones are versatile intermediates for the synthesis of some palladium(II) and platinum(II) compounds (Nakamoto *et al.*, 1970) and other coordination compounds (Ma *et al.*, 1999; Yoshida *et al.*, 2005). We present here the structure characterization of (*Z*)-methyl 3-(2,4-dichlorophenyl)-3-hydroxyacrylate.

The molecular structure (Fig.1) exists in a *cis*-enol form which is stabilized by a strong intramolecular O1—H1···O2 hydrogen bond. The crystal structure (Fig.2) is stabilized by intermolecular C—H···O interactions (Table 1) . The C3—H3···O2 interactions generated zigzag chains along the *c* axis which in turn are linked by C10—H10B···O1 interactions giving sheets parallel to (100).

### Experimental

The title compound was synthesized according to the literature procedure of Wu *et al.* (1997).Single crystals suitable for X-ray diffraction were obtained by slow evaporation of an ethanol solution at room temperature.

### Refinement

All H atoms were detected in a difference map, but all other H-atoms were placed in calculated positions and refined using a riding motion approximation, with C—H=0.93–0.96 Å, with *U*_iso_(H)=1.2 or 1.5*U*_eq_(C); O—H=0.82 Å, with *U*_iso_(H)=1.5*U*_eq_(O).

### Crystal data

**Table:**

C_10_H_8_Cl_2_O_3_	*F*(000) = 504
*M**_r_* = 247.06	*D*_x_ = 1.562 Mg m^−^^3^
Monoclinic, *C**c*	Mo *K*α radiation, λ = 0.71073 Å
Hall symbol: C -2yc	Cell parameters from 5046 reflections
*a* = 15.889 (3) Å	θ = 3.3–27.0°
*b* = 3.8242 (8) Å	µ = 0.60 mm^−^^1^
*c* = 18.204 (4) Å	*T* = 294 K
β = 108.18 (3)°	Prism, colorless
*V* = 1050.9 (4) Å^3^	0.25 × 0.20 × 0.15 mm
*Z* = 4	

### Data collection

**Table:**

Rigaku SCXmini diffractometer	2170 reflections with *I* > 2σ(*I*)
Radiation source: fine-focus sealed tube	*R*_int_ = 0.030
graphite	θ_max_ = 27.5°, θ_min_ = 4.1°
Detector resolution: 13.6612 pixels mm^-1^	*h* = −20→20
CCD_Profile_fitting scans	*k* = −4→4
5011 measured reflections	*l* = −23→23
2371 independent reflections	

### Refinement

**Table:**

Refinement on *F*^2^	Hydrogen site location: inferred from neighbouring sites
Least-squares matrix: full	H-atom parameters constrained
*R*[*F*^2^ > 2σ(*F*^2^)] = 0.034	*w* = 1/[σ^2^(*F*_o_^2^) + (0.0413*P*)^2^ + 0.0839*P*] where *P* = (*F*_o_^2^ + 2*F*_c_^2^)/3
*wR*(*F*^2^) = 0.086	(Δ/σ)_max_ < 0.001
*S* = 1.07	Δρ_max_ = 0.17 e Å^−^^3^
2371 reflections	Δρ_min_ = −0.18 e Å^−^^3^
139 parameters	Extinction correction: *SHELXL97* (Sheldrick, 2008), Fc^*^=kFc[1+0.001xFc^2^λ^3^/sin(2θ)]^-1/4^
2 restraints	Extinction coefficient: 0.0193 (14)
Primary atom site location: structure-invariant direct methods	Absolute structure: Flack (1983), 1177 Friedel pairs
Secondary atom site location: difference Fourier map	Flack parameter: 0.07 (6)

### Special details

**Table:**

Geometry. All e.s.d.'s (except the e.s.d. in the dihedral angle between two l.s. planes) are estimated using the full covariance matrix. The cell e.s.d.'s are taken into account individually in the estimation of e.s.d.'s in distances, angles and torsion angles; correlations between e.s.d.'s in cell parameters are only used when they are defined by crystal symmetry. An approximate (isotropic) treatment of cell e.s.d.'s is used for estimating e.s.d.'s involving l.s. planes.
Refinement. Refinement of *F*^2^ against ALL reflections. The weighted *R*-factor *wR* and goodness of fit *S* are based on *F*^2^, conventional *R*-factors *R* are based on *F*, with *F* set to zero for negative *F*^2^. The threshold expression of *F*^2^ > σ(*F*^2^) is used only for calculating *R*-factors(gt) *etc*. and is not relevant to the choice of reflections for refinement. *R*-factors based on *F*^2^ are statistically about twice as large as those based on *F*, and *R*- factors based on ALL data will be even larger.

### Fractional atomic coordinates and isotropic or equivalent isotropic displacement parameters (Å^2^)

**Table:**

	*x*	*y*	*z*	*U*_iso_*/*U*_eq_	
C1	0.58573 (13)	0.7958 (6)	0.44452 (12)	0.0381 (5)	
C2	0.53832 (13)	0.7161 (6)	0.36792 (12)	0.0392 (5)	
C3	0.57482 (16)	0.7526 (7)	0.30884 (13)	0.0439 (5)	
H3	0.5423	0.6944	0.2583	0.053*	
C4	0.65998 (16)	0.8762 (6)	0.32574 (14)	0.0478 (5)	
C5	0.70941 (16)	0.9602 (7)	0.40023 (15)	0.0520 (6)	
H5	0.7669	1.0447	0.4109	0.062*	
C6	0.67267 (16)	0.9175 (7)	0.45842 (14)	0.0495 (6)	
H6	0.7065	0.9709	0.5088	0.059*	
C7	0.55234 (15)	0.7569 (6)	0.51099 (12)	0.0424 (5)	
C8	0.47153 (15)	0.8564 (6)	0.51265 (13)	0.0424 (5)	
H8	0.4315	0.9548	0.4689	0.051*	
C9	0.44659 (15)	0.8113 (7)	0.58190 (13)	0.0441 (5)	
C10	0.3367 (2)	0.8937 (8)	0.64204 (16)	0.0612 (7)	
H10A	0.3348	0.6501	0.6542	0.092*	
H10B	0.2787	0.9934	0.6319	0.092*	
H10C	0.3776	1.0126	0.6849	0.092*	
Cl1	0.43104 (3)	0.55059 (16)	0.34217 (3)	0.05160 (19)	
Cl2	0.70467 (4)	0.9249 (2)	0.25046 (4)	0.0757 (3)	
O1	0.61264 (12)	0.6173 (6)	0.57216 (10)	0.0629 (5)	
H1	0.5931	0.6120	0.6088	0.094*	
O2	0.49301 (12)	0.6776 (6)	0.64092 (9)	0.0620 (5)	
O3	0.36513 (11)	0.9310 (5)	0.57450 (9)	0.0521 (4)	

### Atomic displacement parameters (Å^2^)

**Table:**

	*U*^11^	*U*^22^	*U*^33^	*U*^12^	*U*^13^	*U*^23^
C1	0.0333 (10)	0.0443 (11)	0.0353 (10)	−0.0018 (9)	0.0086 (8)	−0.0022 (8)
C2	0.0305 (9)	0.0449 (12)	0.0401 (11)	−0.0029 (8)	0.0081 (8)	−0.0011 (9)
C3	0.0384 (10)	0.0573 (13)	0.0350 (12)	−0.0029 (10)	0.0103 (10)	−0.0052 (10)
C4	0.0450 (12)	0.0598 (15)	0.0441 (12)	0.0013 (11)	0.0218 (10)	0.0021 (11)
C5	0.0355 (11)	0.0657 (15)	0.0541 (14)	−0.0083 (10)	0.0130 (11)	−0.0040 (12)
C6	0.0368 (12)	0.0687 (15)	0.0386 (12)	−0.0056 (11)	0.0056 (10)	−0.0073 (10)
C7	0.0412 (11)	0.0494 (12)	0.0329 (11)	−0.0003 (9)	0.0062 (9)	−0.0009 (9)
C8	0.0390 (11)	0.0525 (13)	0.0349 (11)	0.0029 (9)	0.0104 (9)	0.0063 (10)
C9	0.0423 (12)	0.0534 (13)	0.0366 (11)	0.0007 (10)	0.0124 (10)	0.0003 (10)
C10	0.0577 (15)	0.083 (2)	0.0505 (15)	0.0069 (14)	0.0281 (13)	0.0036 (13)
Cl1	0.0372 (3)	0.0734 (4)	0.0422 (3)	−0.0139 (3)	0.0093 (2)	−0.0054 (3)
Cl2	0.0592 (4)	0.1199 (7)	0.0596 (4)	−0.0099 (5)	0.0351 (4)	0.0000 (5)
O1	0.0466 (9)	0.1045 (15)	0.0347 (9)	0.0182 (10)	0.0085 (7)	0.0137 (9)
O2	0.0507 (10)	0.0981 (14)	0.0363 (9)	0.0163 (10)	0.0124 (8)	0.0170 (9)
O3	0.0434 (8)	0.0738 (12)	0.0412 (9)	0.0096 (8)	0.0164 (7)	0.0078 (8)

### Geometric parameters (Å, °)

**Table:**

C1—C2	1.396 (3)	C7—O1	1.333 (3)
C1—C6	1.404 (3)	C7—C8	1.349 (3)
C1—C7	1.472 (3)	C8—C9	1.445 (3)
C2—C3	1.379 (3)	C8—H8	0.9300
C2—Cl1	1.740 (2)	C9—O2	1.210 (3)
C3—C4	1.375 (3)	C9—O3	1.340 (3)
C3—H3	0.9300	C10—O3	1.443 (3)
C4—C5	1.376 (4)	C10—H10A	0.9600
C4—Cl2	1.739 (2)	C10—H10B	0.9600
C5—C6	1.370 (3)	C10—H10C	0.9600
C5—H5	0.9300	O1—H1	0.8200
C6—H6	0.9300		
			
C2—C1—C6	116.5 (2)	O1—C7—C8	122.4 (2)
C2—C1—C7	125.31 (18)	O1—C7—C1	112.18 (19)
C6—C1—C7	118.17 (19)	C8—C7—C1	125.4 (2)
C3—C2—C1	121.98 (19)	C7—C8—C9	120.5 (2)
C3—C2—Cl1	116.21 (16)	C7—C8—H8	119.7
C1—C2—Cl1	121.77 (16)	C9—C8—H8	119.7
C4—C3—C2	119.0 (2)	O2—C9—O3	122.4 (2)
C4—C3—H3	120.5	O2—C9—C8	124.6 (2)
C2—C3—H3	120.5	O3—C9—C8	113.00 (19)
C3—C4—C5	121.3 (2)	O3—C10—H10A	109.5
C3—C4—Cl2	118.42 (19)	O3—C10—H10B	109.5
C5—C4—Cl2	120.24 (19)	H10A—C10—H10B	109.5
C6—C5—C4	119.0 (2)	O3—C10—H10C	109.5
C6—C5—H5	120.5	H10A—C10—H10C	109.5
C4—C5—H5	120.5	H10B—C10—H10C	109.5
C5—C6—C1	122.2 (2)	C7—O1—H1	109.5
C5—C6—H6	118.9	C9—O3—C10	115.47 (19)
C1—C6—H6	118.9		
			
C6—C1—C2—C3	0.4 (4)	C7—C1—C6—C5	179.7 (2)
C7—C1—C2—C3	−178.6 (2)	C2—C1—C7—O1	136.9 (2)
C6—C1—C2—Cl1	178.08 (18)	C6—C1—C7—O1	−42.0 (3)
C7—C1—C2—Cl1	−0.9 (3)	C2—C1—C7—C8	−44.8 (3)
C1—C2—C3—C4	−1.0 (4)	C6—C1—C7—C8	136.3 (3)
Cl1—C2—C3—C4	−178.9 (2)	O1—C7—C8—C9	−0.6 (4)
C2—C3—C4—C5	0.7 (4)	C1—C7—C8—C9	−178.8 (2)
C2—C3—C4—Cl2	−179.34 (19)	C7—C8—C9—O2	−2.1 (4)
C3—C4—C5—C6	0.3 (4)	C7—C8—C9—O3	178.5 (2)
Cl2—C4—C5—C6	−179.7 (2)	O2—C9—O3—C10	0.7 (4)
C4—C5—C6—C1	−1.0 (4)	C8—C9—O3—C10	−179.9 (2)
C2—C1—C6—C5	0.7 (4)		

### Hydrogen-bond geometry (Å, °)

**Table:**

*D*—H···*A*	*D*—H	H···*A*	*D*···*A*	*D*—H···*A*
O1—H1···O2	0.82	1.87	2.592 (3)	146.
C3—H3···O2^i^	0.93	2.48	3.356 (3)	157.
C10—H10B···O1^ii^	0.96	2.57	3.492 (3)	162.

Symmetry codes: (i) *x*, −*y*+1, *z*−1/2; (ii) *x*−1/2, *y*+1/2, *z*.
